# Gender-Specific Determinants and Patterns of Online Health Information Seeking: Results From a Representative German Health Survey

**DOI:** 10.2196/jmir.6668

**Published:** 2017-04-04

**Authors:** Eva Baumann, Fabian Czerwinski, Doreen Reifegerste

**Affiliations:** ^1^ Hanover Center for Health Communication Department of Journalism and Communication Research Hanover University of Music, Drama, and Media Hanover Germany

**Keywords:** health information seeking, social media, gender differences, frequency of seeking, Internet

## Abstract

**Background:**

Online health information-seeking behavior (OHISB) is currently a widespread and common behavior that has been described as an important prerequisite of empowerment and health literacy. Although demographic factors such as socioeconomic status (SES), age, and gender have been identified as important determinants of OHISB, research is limited regarding the gender-specific motivational determinants of OHISB and differences between women and men in the use of online resources for health information purposes.

**Objective:**

The aim of this study was to identify gender-specific determinants and patterns of OHISB by analyzing data from a representative German sample of adults (N=1728) with special attention to access and frequency of use as well as topics and sources of OHISB.

**Methods:**

We employed a 2-step analysis, that is, after exploring differences between users and nonusers of online health information using logistic regression models, we highlighted gender-specific determinants of the frequency of OHISB by applying zero-truncated negative binomial models.

**Results:**

Age (odds ratio, OR for females=0.97, 95% CI 0.96-0.99) and degree of satisfaction with one’s general practitioner (GP) (OR for males=0.73, 95% CI 0.57-0.92) were gender-specific determinants of access to OHISB. Regarding the frequency of OHISB, daily Internet use (incidence rate ratio, IRR=1.67, 95% CI 1.19-2.33) and a strong interest in health topics (IRR=1.45, 95% CI 1.19-1.77) were revealed to be more important predictors than SES (IRR for high SES=1.25, 95% CI 0.91-1.73).

**Conclusions:**

Users indicate that the Internet seems to be capable of providing a valuable source of informational support and patient empowerment. Increasing the potential value of the Internet as a source for health literacy and patient empowerment requires need-oriented and gender-specific health communication efforts, media, and information strategies.

## Introduction

### The Relevance of Health Information Seeking

Patients today are increasingly challenged to make informed choices regarding their health care and to play an active role in health-related decisions [1,2], a change which has been described as *empowerment* [3]. However, a relevant precondition of empowerment is health literacy (ie, the skills and competencies to find and evaluate health information [4-6]). In contrast to the established body of research concerning the concept of health literacy and corresponding measures—which includes a constantly growing body of evidence on the determinants of health literacy and the programs that are effective in enhancing it—research on health information seeking, a behavior closely linked to health literacy, has just began to evolve over the past few years [7]. The dearth of research in this area is all the more astonishing as health information seeking behavior is known to have a strong influence on health-related behavioral intentions, decisions, and outcomes [8].

The Internet represents an increasingly important source of health information [3,9,10], and health is one of the most common topics in online information seeking [9]. Although there is increasing health information available [11], some populations do not sufficiently benefit from the available resources of health information, due to limited access or low media literacy [12]; such population-specific differences raise the risk of increasing health inequalities, commonly referred to as the “digital health divide” [13,14]. In order to improve access to health information [6], we not only need comprehensive research on the gap between *health-onliners* (people who use the Internet to search for health information) and *health-offliners* (people who use channels other than the Internet to search for health information), but also information regarding the determinants of the frequency with which people seek health information online. Although online health information seeking has been analyzed mostly as a binary yes-no outcome, such studies have made only limited contributions to the research about the determinants of the frequency of seeking. Frequency of online health information-seeking behavior (OHISB) is becoming increasingly important as more people use the Internet [9], and an in-depth analysis of the major determinants and outcomes of OHISB is needed.

In light of this clear need—and in parallel to the discourse on the digital divide [14]—the focus of research on OHISB shifts from formerly relevant questions of access to and availability of mere technology toward a deeper understanding of usage frequencies, including demographic, motivational, and health-related factors influencing the frequency of OHISB [15]. Among these factors, gender differences have been frequently reported as relevant for OHISB and health outcomes [9,16], but little is known about the underlying reasons for such differences. Apart from gender differences in general Internet usage [17], reasons for gender differences in OHISB might include the existence of different patterns concerning topics and sources of health information seeking [18] or the lower interest of men in health: Because men tend to be comparably less willing and motivated to engage with health topics [19], they might search for online health information less frequently than do women.

It has been argued that gender differences in OHISB might be concealed by differing motives for seeking health information: Whereas women are more interested in health issues and emotional support, men are more interested in informational support [20]. Men’s higher interest in and earlier acceptance of technology [21] has also resulted in higher mHealth adoption intentions compared with women [22]. Gender differences have also been reported in mobile phone gratifications [23], social media usage [17], and activity in social support groups [20].

Our aim was therefore to understand gender-specific determinants and patterns of OHISB. This understanding will allow us to gain insight into gender-specific preferences regarding content and sources, and to draw conclusions regarding gender-specific targeting strategies for the development of health-related online media. To date, no representative data on gender-specific OHISB for Germany has been analyzed using multivariate statistics [24], making this paper the first such contribution. Our research for this paper investigated the correlates of health-related online information seeking with special regard to gender differences, conducting a secondary analysis of the German Bertelsmann Health Care Monitor 2015. We conclude with a discussion of implications with regard to health communication theory and practice.

### Theories of Health Information Seeking

The models that are frequently used to explain health information seeking—such as the theory of planned behavior (TPB [25]), the theory of motivated information management [26], the risk perception attitude framework [27], or the model of risk information seeking and processing [28]—primarily concentrate on psychological variables (eg, risk perception, subjective norms, control beliefs, or personal experience) or content criteria of the media as determinants of health information-seeking behavior (HISB) [29,30]. As a result, these models and the studies referring to them neglect the direct impact of gender on health information seeking, as well as the related reasons underlying this effect.

Although research has shown that females are more likely to conduct HISB than males, integration of this finding into theory is still lacking [31]. For example, the TPB includes gender as a relevant external variable that influences intentional and attitude-related processes, but the model does not specify the influence of gender [32]; as a result, the TPB only allows researchers to draw limited conclusions about gender-specific health information strategies. One possible explanation for gender-specific patterns of HISB might be found in social role theory [33], which posits that whereas the male gender role casts men as agentic (ie, task-oriented), women are expected to be more socially engaged, with activities such as staying in contact with family members or receiving understanding and feedback from others [23]. These different social roles may contain gender-specific health-related tasks such as care for children or elderly family members, which are in turn associated with an increased demand for health information [12]. These different social roles have also been associated with different use of media channels [17,34] and might be related to different goals for OHISB. In addition to the sociocultural theory, Meyers-Levy and Loken [35] described evolutionary theory, hormone exposure of the brain, and selectivity hypothesis as further theoretical approaches to explain gender differences. These more biological approaches might be especially relevant to explain differences between men and women in the seeking and provision of social support [20], technical affinity [21], motivations for mediated communication [17], and information processing strategies [35].

To date, there has been only minor exploration as to if and how these differences in motives and channel usage are also relevant for online health information seeking. Gaining a better understanding of gender differences in HISB would help health communication scholars to develop gender-specific health communication interventions. Regarding such considerations, our analysis may contribute to the iterative junction of theoretical approaches on HISB and gender differences.

### Gender Differences in General Internet Use and Frequency

Today, the vast majority of the population across Europe and North America has access to the Internet [36], including 86.2% of German residents. Due to the ever-decreasing proportion of Internet nonusers, the discussion on differences between users and nonusers has shifted from access to skills [37]. In general, differences in Internet usage are consistently reported to highly depend on education, age, and gender [38], and socioeconomic status (SES)—a combined measure of education, income, and social position—is strongly correlated with both frequency and patterns of Internet use [39]. Age has been found to be negatively associated with both Internet access and frequency of use [40,41].

Regarding gender differences, findings are somewhat inconsistent, that is, no significant differences in general Internet use have been detected in the United States [40]. The same is true for many other similarly developed countries (eg, Sweden, Norway, the United Kingdom) across Europe, where only minor differences have been found [42]. However, a higher proportion of men than women in Germany reported using the Internet “at least occasionally” (83.0% vs 76.0%, respectively) or “daily” (68.3% vs 58.0%, respectively) [43]; similar numbers have been reported from some other European countries (eg, Austria, Italy, Switzerland), although the significance of these differences has not yet been determined [42]. In addition, gender differences in Internet use might be interacting with age. Although they are evident in older cohorts, they tend to be smaller in younger age groups [7,44]. Men and women seem to differ in both their motivations for and utilization of multiple forms of mediated online communication [17], referring both to topics they search for and to the ways they communicate. These results indicate that women, compared with men, prefer and more frequently engage in interpersonal communication online, using tools such as social networking sites to maintain relationships [45].

### Online Health Information-Seeking Behavior

The Internet’s already-prominent role in HISB continues to increase, that is, in the United States, 59% of the adult population (ie, more than 72% of adult Internet users) seeks online information concerning health topics [9], and the numbers for Germany and other European countries are similar [46]. The Internet is such a popular source of health information primarily because it is an active information channel with a wide range of information on health content, health communities, and health provision [47]. The Internet as a health information source has been found to be especially important for people suffering from chronic diseases [15,48] and for those who are newly diagnosed with a medical condition or health problem [49]. For them, OHISB is a way to obtain more in-depth information, as well as a way to seek out support and contact with other people affected by the same medical condition or diagnosis. This access to social, informational, and emotional support on specific topics then empowers people to manage their health and to take a more active role when interacting with their physicians [3,50,51]. However, significant disparities still exist regarding access to and the ability to process health information online, with older and less educated people being less likely to take advantage of this resource [2,16,52,53].

Gender differences have not only been reported for general Internet use, but also for general health-related behaviors and outcomes, with men having higher mortality and morbidity rates, engaging in more risky behaviors (eg, smoking, alcohol abuse), and taking part in fewer health-promoting behaviors than women [54]. Men also tend to underestimate their health risks, which can lead to avoidance and reactance toward traditional risk information messages; however, despite these differences, little is known about effective gender-specific health communication strategies [55].

With regard to gender-specific HISB, many studies show that women are more engaged in health information seeking in general, as well as on the Internet, specifically. Being female is among the strongest predictors of conducting OHISB [15,48,49]. Whereas women report to be more interested in health information and show more active search activities [56], men are less likely to read health information [57]. This gender gap in OHISB was found to be stable over time when analyzing six waves of Health Information National Trends Survey (HINTS)–data from 2002 to 2013 [31], and was also proven in a robust meta-analysis on US adults [7]; in contrast, recent research from a German sample indicated only a minor gender gap in frequency of OHISB, which did not reach statistical significance [24]. Results have also been inconsistent regarding the channels utilized: Bidmon and Terlutter [24] found that women used health forums, blogs, and search engines as sources more frequently than men, whereas men used apps for OHISB more frequently. In contrast, other studies have reported that men use health-related apps [58] and track health-related indicators as often as women do [9].

Our main goal was to analyze gender-specific determinants and patterns of OHISB. Our first question was which sociodemographic—including gender—and health-related user characteristics explain general utilization of health information on the Internet (RQ1). The second question was whether—among those who use the Internet for health-related purposes—the same factors determine the frequency of OHISB; to address this question, we ask which sociodemographic and health-related user characteristics—in relation to gender—explain the frequency of OHISB (RQ2).

Among the health-onliners, we are also interested in the gender-specific health-related topics they are most interested in and the online media they prefer to use as sources of health information (RQ3).

## Methods

### Data Collection and Sample Size

Data were taken from the Bertelsmann Health Care Monitor 2015, a representative national German health survey (available as open access files) conducted by the Bertelsmann Foundation in cooperation with the Barmer GEK, a statutory health insurance (see [59] for data and further information). This survey assesses health-related knowledge, attitudes, and behaviors, and is similar to the annual American HINTS [12] or the Pew polls [9]. The Bertelsmann Health Care Monitor has been conducted annually via mail since 2001. Its average response rate is about 70%, and it has been established as an important data source in the field of health research, with a number of key publications based on it [60]. The basic population for the survey consists of persons living in private households, aged 18-79 years, in Germany. Samples are drawn from the GfK (German Association for Consumer Research) Mail Panel and the sample is representative concerning gender, age, the German federal states, income, and education compared with the data of the Statistical Yearbook of Germany [61,62]. Although the 2015 Bertelsmann Health Care Monitor comprised 1728 German adults aged 18-79 years, for this study, we excluded respondents who reported not to use the Internet at all, since our main interest is to explain OHISB. Thus, the remaining sample size was N=1219.

The excluded Internet nonusers showed statistically significant differences for several demographic criteria: They were much older (mean 64.7 years, SD 12.1) than the Internet users (mean 46.0, SD 15.0) with a higher proportion of female respondents (59.9% [299/499] vs 50.04% [610/1219]) and lower SES (31.2% [150/481] vs 15.86% [177/1116]). These findings confirmed prior research concerning demographic determinants of general Internet use [39,40,43].

### Measures

#### Online Health Information Seeking Behavior (OHISB)

Our main analyses were based on participant responses to the question “How many times did you use the Internet for seeking health information within the last 12 months?” Answers ranged from 0 to 130 with a mean of 4.37 (SD 9.44); answers were strongly right skewed (skewness=5.85, SE 0.07). To address our first analytical goal of uncovering the gender-specific determinants of utilization of OHISB, we created a dummy variable to separate health-offliners (OHISB=0) from health-onliners (OHISB≥1). To meet our second goal of assessing gender-specific determinants of the frequency of OHISB, we left the responses on their original scale but excluded the health-offliners from the analysis, as they showed no variance in their HISB frequency. This resulted in a final sample of 643 health-onliners.

The third objective—assessing gender differences in health-related topics and information sources—was achieved by analyzing the frequencies of the health-related topics and websites the health-onliners used. Respondents were asked to select the topics on which they searched for or received information from a list of 14 items. These items ranged from very specific (eg, “drugs and their pharmacological interactions”) to more general (eg, “fitness, well-being”) topics. These items were then grouped into three categories by content: “disease and health care,” “health care policy and health care system,” and “health and well-being.”

Respondents were then asked to select the sources they used when conducting OHISB, that is, they were given the 10 items to choose from popular sources (eg, “online dictionary”) and more specific sources (eg, “websites of noncommercial health organizations”).

#### Predictor Variables of Online Health Information Seeking Behavior (OHISB)

##### Demographic Variables

Participants were asked to provide their age in years and gender (female or male), whereas SES was assessed by summing up participants’ responses on their education, occupation, and income (weighted by household size) to a score ranging between 3 and 27 following the standard procedure for the Health Care Monitor [61]. Due to the application of conventional formats, data on SES were transformed from the original 27-point scale to a 3-point scale analog indicating “low,” “medium,” and “high” SES.

##### Variable Related to General Internet Use

The frequency of general Internet usage was measured using a 3-point ordinal scale of “at least sometimes per month,” “several times per week,” or “daily.”

##### Health-Related Variables

Patient status was measured using a 4-point scale ranging from 1 (“currently not affected”) to 4 (“chronically ill”). We classified the responses from “mildly or not affected” to “severely or chronically ill,” because OHISB patterns of healthy and mildly affected respondents should be quite similar, whereas severely or chronically affected people were expected to show fundamentally different patterns. Participants’ perceived relevance of understanding somatic processes, their health-consciousness, and the satisfaction with their GP were all measured using 5-point scales, that is, to measure health-consciousness, participants were asked how much attention they generally paid to their health, which they rated from 1 (“Generally, I don’t take care of my health”) to 5 (“Generally, I take good care of my health”). Satisfaction with their GP was scored from 1 (“very dissatisfied”) to 5 (“very satisfied”), their perceived relevance of understanding somatic processes was assessed by their degree of agreement—from 1 (“totally disagree”) to 5 (“totally agree”)—concerning the statement that “patients diagnosed with an illness should understand exactly what is going on.”

The extent to which respondents reported being interested in information concerning health topics in general was originally measured on a 3-point scale indicating weak, medium, or strong interest. We transferred these answers into a dummy variable, contrasting “low or medium level of interest” with “high level of interest” to create reasonably equal group sizes (n=739 and n=450, respectively). Looking at health-onliners only, their motivations to conduct HISB were assessed using 12 dummy indicators covering a broad range of potential goals (eg, “to find general health information about health risks and diseases” or “determining the best treatment options”). On the basis of social support theory [63], we categorized these 12 indicators into 3 indices representing aspects of “esteem support” (5 items), “informational support” (5 items), and “emotional support” (2 items). The more items participants agreed within each index, the higher their score (one point per item).

Items are given in Multimedia Appendix 1.

### Statistical Analysis

To answer RQ1, a logistic regression model was conducted to analyze the influence of sociodemographic, motivational, and health-related factors on differences between health-onliners and health-offliners. Regarding RQ2, Poisson regression models are traditionally used to model data like the frequency of OHISB, as such models are suited to fulfilling the technical needs of an outcome consisting of positive integers. However, the application of Poisson models requires a data structure that is seldom found in reality, that is, the mean is equal to the variance [64,65]. As the variance in real data is often much bigger than the mean, “overdispersion” tends to occur, leading to biased variance estimates and associated inferential problems [66,67]. This was certainly the case for our data, as the variance (136.9) is about 16 times greater than the mean (8.32), indicating severe problems due to strong overdispersion. Furthermore, since we excluded all health-offliners (with an OHISB frequency=0), our data contain no zeros, and the application of a standard negative binomial model—which tries to predict zeros—should therefore be avoided [68]. We therefore conducted zero-truncated negative binomial regression models to explain the frequency of health information searching. Missing values were deleted listwise for all multivariate analyses. All analyses were conducted using SPSS 22 (IBM Corporation) except the zero-truncated negative binomial models, which were estimated using Stata 11.2 (Stata Corp LLP).

## Results

### Sample Description

Among all of the 1219 participants who used the Internet, 643 (52.75%; *health-onliners*) searched for health information online and 576 did not (47.25%; *health-offliners*; see Table 1). The health-offliners showed no significant differences from health-onliners regarding age and gender, but significantly fewer health-offliners had high SES (*P*=.001). Health-onliners rated their own health status more often as “chronically or severely ill” (n=142 respondents or 22.1% vs n=103 or 17.8% in health-onliners), but the difference was not significant (*P*=.06). Health-onliners’ satisfaction with their GP was slightly lower than health-offliners’ (mean 4.02, SD 0.86 vs mean 4.13, SD 0.86, respectively; *P*=.03). Significant differences between health-onliners and health-offliners were found for several health-related variables, with perceived relevance of understanding somatic processes (mean 4.34, SD 0.83 vs mean 4.16, SD 0.90; *P*<.001) and health-consciousness (mean 3.59, SD 0.71 vs mean 3.34, SD 0.81; *P*<.001) higher among the health-onliners. Additionally, health-onliners were more likely to report being strongly interested in information concerning health topics (n=297 or 46.2% vs n=164 or 28.5%; *P*<.001) and generally used the Internet more often than health-offliners, with 65.3% versus 50.5% (corresponding to n=420 vs n=291 respondents) reporting using the Internet “daily” (*P*<.001).

**Table 1:** Sample characteristics of health-onliners and health-offliners.

Variable		Total sample	Health-onliners	Health-offliners	Difference onliners versus offliners		
		n=1219	n=643	n=576	*F* or chi-square statistics^a,b^	Degree of freedom	*P* value
**Age**							
	Range	18-79	18-79	18-78			
	Mean (SD^c^)	46.02 (14.96)	45.35 (14.55)	46.78 (15.38)	2.812^a^	1	.09
**Gender, n (%)**							
	Female	610 (50.04)	338 (52.6)	272 (47.2)	3.5^b^	1	.06
	Male	609 (49.96)	305 (47.4)	304 (52.8)			
**Socioeconomic status, n (%)**							
	Low	177 (15.86)	79 (13.4)	98 (18.6)			
	Medium	658 (58.96)	339 (57.5)	319 (60.6)	13.1^b^	2	.001
	High	281 (25.18)	172 (29.2)	109 (20.7)			
**General Internet use, n (%)**							
	At least sometimes per month	189 (15.50)	68 (10.6)	121 (21)			
	Several times per week	319 (26.17)	155 (24.1)	164 (28.5)	34.9^b^	2	<.001
	Daily	711 (58.33)	420 (65.3)	291 (50.5)			
**Patient status, n (%)**							
	Chronically or severely ill	243 (20.05)	141 (22.1)	102 (17.8)	3.4^b^		.06
	Mildly or not affected	969 (79.95)	498 (77.9)	471 (82.2)		1	
Perceived relevance of understanding somatic processes^d^, mean (SD)		4.25 (0.86)	4.34 (0.83)	4.16 (0.90)	13.012^a^	1	<.001
**Interest in information concerning health topics, n (%)**							
	Low or medium	739 (62.15)	336 (53.8)	403 (71.5)	39.5^b^	1	<.001
	Strong	450 (37.85)	289 (46.2)	161 (28.5)			
**Goals for HISB^e,f^**, **mean (SD)**							
	Esteem support	0.24 (0.26)	0.24 (0.26)	-	N/A^g^		
	Emotional support	0.06 (0.20)	0.06 (0.20)	-	N/A		
	Informational support	0.37 (0.23)	0.37 (0.23)	-	N/A		
Health-consciousness^h^, mean (SD)		3.47 (0.77)	3.59 (0.71)	3.34 (0.81)	33.431^a^	1	<.001
Satisfaction with general practitioner^i^, mean (SD)		4.07 (0.86)	4.02 (0.86)	4.13 (0.86)	4.492	1	.03

^a^*F* values derived from analysis of variance (ANOVA) for continuous variables.

^b^Chi-square values derived from chi-square test for shares.

^c^SD: standard deviation.

^d^Scale ranges from 1 (“strongly disagree”) to 5 (“strongly agree”).

^e^HISB: health information-seeking behavior.

^f^Scale ranges from 0 (“no” for all items of the scale) to 1 (“yes” for all items of the scale).

^g^N/A: not applicable.

^h^Scale ranges from 1 (“Generally, I don’t take care of my health”) to 5 (“Generally, I take good care of my health”).

^i^Scale ranges from 1 (“very unsatisfied”) to 5 (“very satisfied”).

### RQ1: Using the Internet to Search for Health Information

The results of the logistic regression models are depicted in Table 2 and the strength of the association between each predictor variable and the outcome is expressed in form of odds ratio (OR), which indicates the expected change in the odds to observe the outcome (ie, to be a health-onliner) when the respective predictor changes by one unit. There is no evidence for a main effect of gender (OR 1.21, 95% CI 0.90-1.61), but age and SES were significant predictors of being a health-onliner or health-offliner. However, a 1-year increase in age was associated with a decreased OR of being a health-onliner for women by the factor 0.97 (OR 0.97, 95% CI 0.96-0.99) and in the total sample (OR 0.99, 95% CI 0.98-0.997), but not for men. In contrast, a high SES was associated with significantly increased odds of going online for health-related information only for male respondents (OR 1.97, 95% CI 1.06-3.68) and in the combined model, that is, the whole sample of male and female respondents (OR 1.82, 95% CI 1.15-2.88), but not female respondents.

A higher frequency of general Internet use was associated with a nearly triple-increase in the odds of being a health-onliner (OR for “daily” use=2.91, 95% CI 1.92-4.41), with the slightly stronger effects for female (OR 3.23, 95% CI 1.86-5.59) than for male respondents (OR 2.50, 95% CI 1.30-4.78).

Persons who were chronically ill or severely affected by health problems were significantly more likely to be health-onliners, but only if they were women (OR 2.12, 95% CI 1.28-3.53). A similar relationship was found between perceived relevance of understanding somatic processes and HISB, that is, for women, a one-point increase in the perceived importance of health literacy was associated with an OR 1.39 (95% CI 1.09-1.78) of being a health-onliner, whereas men had only a moderately heightened OR that did not reach significance. Although both male and female respondents appeared to be significantly influenced by having general interest in information on health topics, this impact was much stronger among female participants (OR_women_ 2.07, 95% CI 1.36-3.14; OR_men_ 1.70, 95% CI 1.09-2.63, respectively).

Degree of health-consciousness was associated with significantly increased OR for men (OR 1.46, 95% CI 1.10-1.94) and for the combined model (OR 1.33, 95% CI 1.10-1.61), but not for women alone. Higher satisfaction with one’s GP had a negative effect on the odds that men would seek health information online, that is, be health-onliners (OR 0.73, 95% CI 0.57-0.92).

The Hosmer-Lemeshow test inform on the proper classification of all cases included and gives a chi-square value of 11.5 (df=8; *P*=.17) for the total subsample. Both *P* values for the gender-specific models are also nonsignificant, what confirms no major differences between predicted and observed classification of cases [69]. Consistently, the overall model-fit is quite well, as indicated by the goodness of fit test comparing each full model with the empty model and yielding significant results in all three cases. The explained variance also indicated the existence of gender differences. Comparing men and women, the logit model better fits the data of female respondents: whereas Nagelkerke *R* ² increased to 19.2% for women, it could only explain 13.7% of outcome variance (ie, whether the respondent was a health-onliner) for male respondents.

**Table 2:** Results of the logistic regression models predicting online health information-seeking behavior.

Determinants		Total (n=950)^a^		Male (n=463)		Female (n=487)	
		OR^b^ (95% CI)	*P*	OR (95% CI)	*P*	OR (95% CI)	*P*
Age		0.99 (0.98-1.00)	.01	0.99 (0.98-1.01)	.46	0.97 (0.96-0.99)	.002
Gender (Ref: male)		1.21 (0.90-1.61)	.21	-^c^	-	-	-
**Socioeconomic status** (Ref: low)			.01		.02		.57
	Medium	1.13 (0.77-1.66)	.54	1.07 (0.62-1.86)	.80	1.16 (0.67-2.01)	.59
	High	1.82 (1.15-2.88)	.01	1.97 (1.06-3.68)	.03	1.46 (0.72-2.93)	.29
**General Internet use** (Ref: at least sometimes per month)			<.001		.005		<.001
	Several times per week	1.57 (1.02-2.41)	.04	1.46 (0.72-2.94)	.29	1.70 (0.99-2.94)	.06
	Daily	2.91 (1.92-4.41)	<.001	2.50 (1.30-4.78)	.006	3.23 (1.86-5.59)	<.001
Patient status: chronically or severely ill (Ref: mildly or not affected)		1.56 (1.11-2.19)	.01	1.22 (0.76-1.95)	.42	2.12 (1.28-3.53)	.004
Perceived relevance of understanding somatic processes^d^		1.27 (1.08-1.50)	.005	1.22 (0.97-1.53)	.10	1.39 (1.09-1.78)	.008
Strongly interested in information concerning health topics (Ref: weakly or not interested)		1.89 (1.40-2.54)	<.001	1.70 (1.09-2.63)	.02	2.07 (1.36-3.14)	.001
Health-consciousness^e^		1.33 (1.10-1.61)	.004	1.46 (1.10-1.94)	.008	1.24 (0.95-1.62)	.11
Satisfaction with general practitioner^f^		0.82 (0.70-0.96)	.02	0.73 (0.57-0.92)	.008	0.91 (0.72-1.14)	.40
Constant		0.31	.000	0.35	.006	0.35	.002
Hosmer-Lemeshow test (chi-square, df; *P*)		11.5, 8; .17		12.2, 8; .14		4.7, 8; .79	
Goodness of fit^g^ (chi-square, df; *P*)		116.3, 11; <.001		50.2, 10; <.001		75.5, 10; <.001	
Nagelkerke *R* ²		.154		.137		.192	

^a^The difference between the number of total cases included in the descriptive section and in the logit models is due to the listwise exclusion of missing cases.

^b^OR: odds ratio.

^c^The dash indicates the absence of the variable “gender” in both gender-specific models.

^d^1 (“strongly disagree”) to 5 (“strongly agree”).

^e^1 (Generally, I don’t take care of my health” to 5 (“Generally, I take good care of my health”).

^f^1 (“very unsatisfied”) to 5 (“very satisfied”).

^g^ (−2 Log L compared with −2 Log L of the empty model).

### RQ2: Frequency of Online Health Information-Seeking Behavior (OHISB)

The results of the zero-truncated negative binomial regression models are shown as incidence rate ratio (IRR) in Table 3. The name of the measure has changed to IRR, because the outcome now reflects the number of incidences (Internet access events with the purpose to conduct OHISB) observed in the last year, but the interpretation remains analog to OR, as pointed out above. Focusing only on health-onliners, SES turned out not to be a relevant predictor for higher frequencies of information seeking. Analogous to the results for access to online health information (see Table 2), increasing age was also significantly associated with women’s OHISB frequency (IRR 0.99, 95% CI 0.975-0.996). Since the effect on this outcome is multiplicative, a 1-year increase thus leads to a predicted OHISB frequency, which is decreased by a factor of 0.99. There was no influence of gender on the frequency of OHISB for the total sample (IRR 1.00, 95% CI 0.99-1.01), but differences were again found in the patterns of influences on women versus men.

Although the effects of higher frequencies of general Internet use are similar in size and *P* values in the combined model, the gender-specific models revealed differences between men and women: whereas daily use of general Internet was only associated with a significant increase in OHISB frequency in males (IRR 2.49, 95% CI 1.43-4.35), using the Internet “several times per week” was significant only for female respondents (IRR 1.54, 95% CI 1.01-2.35).

Being a patient with a chronic or severe disease was a positive predictor of OHISB frequency (IRR 1.57, 95% CI 1.26-1.95) regardless of gender, as the estimated IRRs do not differ substantially between men and women. Respondents who reported being strongly interested in information concerning health topics were much more likely to seek out information online more frequently (IRR 1.45, 95% CI 1.19-1.77), and gender played a much weaker role than health status.

Self-reported health-consciousness in the zero-truncated negative binomial regression models was—as compared with the findings from the logit models predicting the utilization of the Internet for health information purposes—not associated with significant effects. In contrast, perceived relevance of understanding somatic processes had the opposite effect on OHISB frequency of that predicted by the logit models: In the zero-truncated negative binomial regression models, belief in health literacy became a significant negative predictor, but for men only (IRR 0.81, 95% CI 0.68-0.96). Satisfaction with one’s GP changed from being a significant factor only for males to being a significant factor only for females, the latter now with a strong negative effect (IRR 0.75, 95% CI 0.65-0.88), whereas men’s frequency of OHISB seems to be statistically unrelated to their degree of satisfaction.

Some of the three sum indices representing different goals of OHISB showed strong explanatory potential: whereas *esteem support* seems to be an important motivational factor only for women (IRR 2.22, 95% CI 1.30-3.79), *informational support* was associated with a quadrupled OHISB frequency per point for women (IRR 4.03, 95% CI 2.17-7.49), and with a slightly weaker effect for men (IRR 2.56, 95% CI 1.34-4.90). The goal of *emotional support* had no influence on respondents’ OHISB frequency.

**Table 3:** Results of the zero-truncated negative binomial regression models on the frequency of online health information-seeking behavior (OHISB).

Determinants		Total (n=510)^a^				Male (n=241)				Female (n=269)			
		IRR^b^ (95% CI)		*P*		IRR (95% CI)		*P*		IRR (95% CI)		*P*	
Age		0.99 (0.982-0.998)		.01		1.00 (0.99-1.01)		.58		0.99 (0.975-0.996)		.009	
Gender (Ref: male)		0.99 (0.82-1.22)		.99		-^c^		-		-		-	
**Socioeconomic status** (Ref: low)													
	Medium		1.06 (0.79-1.41)		.71		1.01 (0.66-1.55)		.97		0.93 (0.63-1.36)		.70
	High		1.25 (0.91-1.73)		.17		1.14 (0.72-1.79)		.57		1.14 (0.72-1.81)		.56
**General Internet use** (Ref: at least sometimes per month)													
	Several times per week		1.60 (1.12-2.27)		.009		1.72 (0.94-3.16)		.08		1.54 (1.01-2.35)		.04
	Daily		1.67 (1.19-2.33)		.003		2.49 (1.43-4.35)		.001		1.28 (0.84-1.96)		.25
Patient status: chronically or severely ill (Ref: mildly or not affected)		1.57 (1.26-1.95)		<.001		1.67 (1.22-2.29)		.001		1.43 (1.07-1.91)		.02	
Perceived relevance of understanding somatic processes^d^		0.90 (0.80-1.01)		.06		0.81 (0.68-0.96)		.02		0.96 (0.82-1.13)		.65	
Strongly interested in information concerning health topics (Ref: weekly or not interested)		1.45 (1.19-1.77)		<.001		1.46 (1.09-1.97)		.01		1.42 (1.10-1.83)		.01	
**Goals of OHISB^e^**													
	Esteem support		1.91 (1.28-2.83)		.001		1.49 (0.82-2.72)		.19		2.22 (1.30-3.79)		.004
	Emotional support		0.90 (0.57-1.43)		.65		1.13 (0.53-2.40)		.75		0.83 (0.46-1.49)		.52
	Informational support		3.12 (1.97-4.96)		<.001		2.56 (1.34-4.90)		.004		4.03 (2.17-7.49)		<.001
Health-consciousness^f^		1.10 (0.97-1.26)		.14		1.70 (0.89-1.29)		.49		1.08 (0.91-1.30)		.38	
Satisfaction with general practitioner^g^		0.86 (0.77-0.96)		.007		1.09 (0.93-1.28)		.29		0.75 (0.65-0.88)		<.001	
Constant		0.47		.04		0.32		.36		0.65		.01	

^a^The difference between the total number of cases included in the descriptive section and in the models depicted in this table is due to the listwise exclusion of missing cases.

^b^IRR: incidence rate ratio.

^c^The - indicates the absence of the variable “gender” in both gender-specific models

^d^From 1 (“strongly disagree”) to 5 (“strongly agree”).

^e^OHISB: online health information-seeking behavior.

^f^From 1 (“Generally, I don’t take care of my health”) to 5 (“Generally, I take good care of my health”).

^g^From 1 (“very unsatisfied”) to 5 (“very satisfied”).

### RQ3: Topics and Sources of Online Health Information-Seeking Behavior (OHISB)

**Figure 1:**
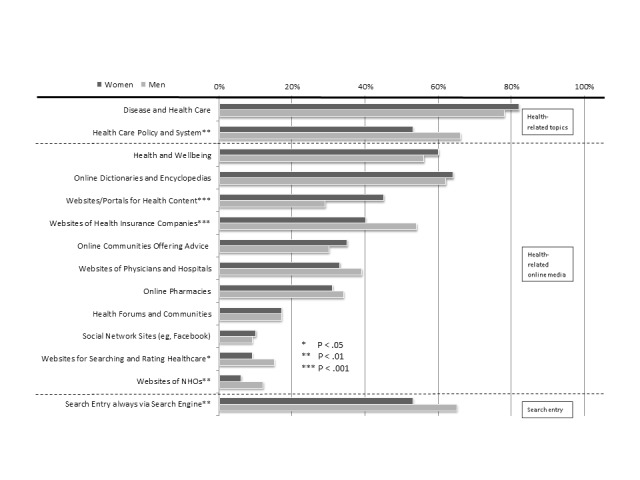
Gender differences in online health information-seeking behavior (OHISB) concerning topics and sources of online communication. NHOs: Noncommercial health organizations (total n=640).

Figure 1 illustrates which health topics women and men are interested in and which online sources they use when they search for health information. Diseases and health care are of particularly interest for about 79.7% of the health-onliners (n=510 of 640 respondents), health and wellbeing are also relevant topics for both women and men. The Internet seems to serve as a source of specific information that is sought primarily using search engines and online dictionaries.

Online media offering the opportunity to share information and to interact with others, specifically, online health communities and social networking sites are not yet established as a means of OHISB in the broad public, with an overall usage of 17.0% and 9.7% (corresponding to 109/640 and 62/640 respondents), respectively. In some cases, we can detect significant differences in issue- and channel-related preferences between women and men, that is, in general, men focus more on topics concerning health care policy and systems (66.4%, 202/304 males vs 53.0%, 178/336 females; *P*=.001), and visit the websites of health insurance companies (53.6%, 163/304 males vs 39.6%, 133/336 females; *P*<.001) and noncommercial health organizations (12.2%, 37/304 men vs 6.0%, 20/336 women; *P*=.006) more frequently than women do. Women reported significantly more usage of websites or portals for health content (44.6%, 150/336 female respondents vs 28.9%, 88/304 males; *P*<.001).

## Discussion

### Principal Findings

Despite the fact that men and women reported equal access to online health information, our data indicate that OHISB should be explained using gender-specific models, to account for several significant gender differences among health-onliners. Dissatisfaction with primary care seems to more often trigger women to seek patient esteem support through online health information seeking; OHISB might therefore serve a compensatory function. These and additional results—particularly regarding gender differences, implications, and methodical limitations—are discussed and compared with international data.

Our results indicate that SES and age remain relevant barriers to general access to health information on the Internet, but only for specific genders. We found increasing age to be significantly associated with both access to and frequency of OHISB for women only, thus enhancing understanding of the gender-specificity of the well-established negative correlation between age and OHISB [7]. In contrast, we found high SES to be associated with increased odds of conducting OHISB among male respondents only; among females, SES had no significant effects on neither the use nor frequency of searching. This finding is partly consistent with former studies: whereas some researchers have reported no significant effect of higher educational levels on the frequency of OHISB [15], others found strong associations between both respondents’ income and educational level on the likelihood of using the Internet to find out more about a medical condition [9].

Higher frequencies of general Internet use revealed to be consistently associated with more frequent OHISB [49]. We found a stronger effect of daily Internet use on the frequency of OHISB for men, whereas women’s OHISB seems to be only slightly influenced by their general Internet use. This specific association lacks direct comparability to former results, although Renahy and colleagues [15] found a positive effect of more frequent Internet use on the frequency of OHISB, with no gender differences.

The effect of being severely or chronically ill affected OHISB differently for different groups. Only severely ill women, not men, were significantly more likely to be health-onliners, consistent with findings from a French study [15]. In contrast, the impact of patient status on the frequency of OHISB was slightly stronger for male respondents. We tentatively interpret these differences as supporting the 2-step data analysis strategy we chose. These results offer new insights into the relationship between patient status and OHISB when compared with existing nongender-differentiated findings [48,49].

The association between OHISB and related online activities and interests (eg, buying drugs and other health-related products online) that indicate a high level of interest in health information is neither surprising nor new, as this has been reported in both an analysis of cross-sectional data from 7 European countries [3] as well as in US data [49]. However, little evidence has been produced to date on the influence of perceived relevance of understanding somatic processes on OHISB. To our knowledge, only one study has investigated a similar variable: Bidmon and Terlutter [24] found that women reported a slightly higher personal disposition of being well-informed as a patient than men, but the difference was not significant and the association with details of OHISB was not explicitly assessed. Even less comparable evidence exists concerning health-consciousness as a predictor of OHISB. This means that our findings indicating that health-consciousness has a significant influence on male utilization of OHISB only may act as a benchmark for future studies.

We found that whereas women are inclined to engage in more frequent OHISB in light of their goals reflecting needs for esteem support and informational support, men tend to be driven more by purely informational motives. This is consistent with another recent finding that women were more likely than men to conduct OHISB for social motives and enjoyment [24]. Comparing the results on our two outcomes of access to and frequency of online health information seeking, the introduction of these indices may have absorbed some explanatory power from health-consciousness.

Our results further indicate that using the Internet can serve a compensatory function, but in different ways for women and men. Whereas a lower satisfaction with one’s GP motivates men to turn to the Internet for health-related purposes (raw usage, independent of the frequency), a lower satisfaction with one’s GP is associated with an increased frequency of OHISB reported by women. These findings are in line with another study reporting that dissatisfied cancer patients seek health information from sources other than their physicians [70], and with a study that found that women engaged in more frequent OHISB when they suspected that their GP was not telling them everything about their health, or when they reported a general preference to wait before going to see a physician [15].

Our findings regarding sources employed for OHISB are partly consistent with a similar study: Females from our sample reported using health content-related websites significantly more often than males, which may reflect the stronger social supportive patterns detected among women [24]. The majority of respondents (n=374 respondents or 59.0%) reported always using a search engine when conducting OHISB; this is consistent with a finding from the United States, in which an even higher proportion of respondents (77%) reported following this strategy [9]. These large proportions indicate that the primary purpose of OHISB is to receive quick and easy access to online health information. Moreover, OHISB reflects a need—especially among men—for health information that is clearly explained and tailored to their specific needs.

### Limitations

The first limitation is that the cross-sectional data used in our analysis do not allow for any causal attributions, even in cases that seem straightforward, such as the effects of age or health status on OHISB.

The second limitation is that outcome operationalization was somewhat explorative, as a well-established, validated scale for assessing access to and frequency of OHISB does not yet exist. Development of a validated measure to assess OHISB is the central precondition of conducting internationally comparable research on this behavior. Such a measure would also complement the valid and reliable measure for assessing eHealth literacy (ie, the ability to seek, find, understand, and appraise health information from electronic sources and apply it to addressing or solving a health problem) that has already been developed [71]. As this inquiry used data from a large-scale representative survey conducted regularly, our results are affected by the typical constraints of a secondary analysis of data that were not primarily collected for the analyzed purposes—the lack of a measure for eHealth literacy [71] is one obvious drawback. Other important characteristics—particularly health status—were measured using one-item self-reports, which offers only a superficial assessment. This criticism can also be applied to other measures of health-related online activities and online usage behavior, as self-reports of online activities often diverge from real behavior [72]. The validity of these findings should be enhanced in future studies by complementarily using objective measures of health-related online activities.

Further limitations include, third, that no differentiation is made between people who are searching for information for themselves and those who are searching for others (“surrogate seekers”). Finally, we only used a binary categorization of men and women, which does not cover all facets of such a complex construct [73]. Our results might have also been influenced by individuals’ gender-role orientation [74]. It might be the case, that women and men only searching for health information both scored rather high on femininity and therefore were more similar than people not seeking for health information. In addition, gender-roles orientations arising from differing social and cultural environments might differentially influence OHISB. Further research should therefore include measures of gender orientation such as the Bem Sex-Role Inventory [75] and samples more diverse in cultural background.

### Conclusions

Our results provide promising and innovative insights into OHISB and indicate that a deeper understanding of OHISB requires differentiating between access to online health information (ie, differentiating between health-onliners and health-offliners) and the frequency of OHISB. This deeper understanding would be particularly valuable for the analysis of what are often subtle gender-based differences. Furthermore, sociodemographic, health-related, and motivational determinants of OHISB should be taken into account when explaining such complex behavior. This recommendation also applies to the associations between skills-related (ie, eHealth literacy) and behavior-related (OHISB) concepts, whose interrelations have yet to be analyzed sufficiently [76].

Overall, although users indicate that the Internet is capable of providing a valuable source of informational support and esteem support, gender-specific, user-oriented sources and empowerment-strategies should be developed to increase the benefits of OHISB. This may include enlisting the support of health care providers to supply patients with health information sources that offer evidence-based, transparent, and credible information. To close the gap in OHISB due to age and SES, such resources might, for example, reduce the complexity of the language and enhance the understandability of the health information offered. Gender-specific determinants and patterns in information-seeking behavior should also be taken into account in theories of health information seeking and in the provision of online health information by offering information in accordance with male and female preferences regarding goals, sources, and topics. For example, men’s technical affinity might be used as a pathway in health communication to raise their interest in health content about diseases and well-being [77], whereas women’s need for emotional support might be met with communication in online communities via social network sites [20].

## Appendix

Multimedia Appendix 1 Items.

## Abbreviations

GP: general practitioner
HINTS: Health Information National Trends Survey
HISB: health information-seeking behavior
IRR: incidence rate ratio
OHISB: online health information-seeking behavior
OR: odds ratio
SES: socioeconomic status
TPB: theory of planned behavior
