# Supplementary material for: Gender-Specific Determinants and Patterns of Online Health Information Seeking: Results From a Representative German Health Survey
Source: J Med Internet Res. 2017 Apr 4;19(4):e92. doi: 10.2196/jmir.6668 (PMC5395693; doi:10.2196/jmir.6668)
Supplement: Multimedia Appendix 1 [file jmir_v19i4e92_app1.pdf]

	Questions/Items (Scales) in English	Items in German
**Variables of OHISB**		
Access to Internet	Did you use the Internet to access health information within the last 12 months? (Yes, No)	Haben Sie in den letzten 12 Monaten das Internet auch zur Suche nach gesundheitlichen Informationen genutzt? (Ja, Nein)
Frequency of general internet usage	How often do you use the internet?<br>(Daily, several times per week, sometimes per month or less, never)	Wie oft gehen Sie ins Internet?<br>(täglich, mehrmals in der Woche, einige Male im Monat oder noch seltener, nie)
Frequency of OHISB	How many times did you use the Internet for seeking health information within the last 12 months?<br>about__times	Wie oft haben Sie in den letzten 12 Monaten das Internet zur Suche nach gesundheitlichen Informationen genutzt?<br>etwa___Mal
Health topics	About which topics did you search or receive health information? (Please mark with a cross)<br>disease/health care<br>health care policy/health care system<br>health and well-being	Zu welchen Themen haben Sie in den letzten 12 Monaten Informationen gesucht bzw. erhalten?(Bitte alles Zutreffende ankreuzen)<br>Krankheit/Gesundheitsversorgunge<br>Gesundheitspolitik/-system<br>Gesunde Lebensweise
Health sources	Which of the following Internet sources about health topics did you use? (Please mark with a cross)<br>Online dictionaries, Websites for health content, websites of health insurances companies, online communities offering advice, social networks sites, health communities, websites of physicians, websites of non-commercial health organizations, blogs, medical online support, websites for rating healthcare, online pharmacies,	Welche der folgenden Internet-Angebote haben Sie rund um das Thema Gesundheit und Krankheit schon einmal genutzt? (Bitte alles Zutreffende ankreuzen)<br>Gesundheits-Infos aus Online-Lexika, Internetseiten von Krankenkassen, Gesundheitsportale, Ratgeber-Communities, soziale Netzwerke, Gesundheitsforen, Webseiten von Ärzten, Webseiten gemeinnütziger Gesundheitsorganisationen, Blogs zu Gesundheitsthemen, Medizinische Online-Beratung, Bewertungsportale, Online-Apotheke
**Potential Determinants of**		

OHISB		
Health consciousness	How much attention do you pay to your health?<br><br>(very much, much, moderate, less, none)	Wie stark achten Sie im Allgemeinen auf Ihre Gesundheit? (sehr stark, stark, mittelmäßig, weniger stark, gar nicht)
Interest in health topics	Which of the following statements would you mostly agree with?<br><br>I try to get much information about health topics related to me.; I do not look for health topics intentionally, but I am interested; I am not very interested in health topics.	Welcher der folgenden Aussagen würden Sie am ehesten zustimmen?<br>Ich versuche, viele Informationen über Gesundheitsthemen zu bekommen, die mich betreffen.<br>Ich achte nicht speziell auf Informationen über Gesundheitsthemen, interessiere mich aber dafür.<br>Ich bin nicht sehr an Informationen über Gesundheitsthemen
Satisfaction with general practitioner	How satisfied are you with your general practioner?<br><br>(very satisfied, unsatisfied)	Wie zufrieden sind Sie insgesamt mit Ihrem Hausarzt und seiner Praxis? (vollkommen zufrieden, unzufrieden)
Perceived relevance of understanding of somatic processes	Patients diagnosed with an illness should understand exactly what is going on. (totally disagree, totally agree)	Was genau bei einer Krankheit im Körper passiert, sollte man als Patient vollständig verstehen. (stimme überhaupt nicht zu/stimme völlig zu)
Health status	How would you describe your health status?<br><br>(bad, less good, good, very good, excellent)	Wie würden Sie Ihren Gesundheitszustand im Allgemeinen beschreiben?<br><br>(schlecht, weniger gut, gut, sehr gut, ausgezeichnet)
Patient status	What applies to you at the moment?<br><br>(currently not affected/without health problems, mildly acute, chronically ill or severely acute)	Was trifft zurzeit auf Sie zu?<br><br>(ich bin gesund / bin ohne gesundheitliche Probleme,<br>ich bin akut leicht erkrankt,<br>ich bin akut schwer erkrankt,<br>ich bin chronisch krank)
Motives for OHISB	When looking for online health information, which aims did you pursue?<br><br>I wanted to …<br><br>Esteem support (5 items)<br><br>…be better prepared for the talk with	Wenn Sie jetzt einmal nur an die Situationen denken, in denen Sie in Gesundheits- oder Krankheitsfragen das Internet genutzt haben, welche Ziele haben Sie da verfolgt?<br><br>Ich wollte …<br><br>Esteem support

	the doctor.<br><br>…understand information of the doctor.<br><br>…check information of my doctor.<br><br>…find additional information to suggestions of the doctor.<br><br>…help myself with an acute health problem.<br><br>Informational support (5 items)<br><br>…find general health information about health risks and disease, determining the best treatment options.<br><br>…find specific information about healthcare and health insurance.<br><br>…find the best health care facilities.<br><br>…tips for healthier lifestyle.<br><br>…give advice to family and friends.<br><br>Emotional support (2 items)<br><br>…be not alone, receive support from others.<br><br>…exchange experiences with others.	…auf das Gespräch mit einem Arzt oder Therapeuten besser vorbereitet sein<br><br>…die Informationen eines Arztes oder Therapeuten nachträglich klären und besser verstehen.<br><br>…Behandlungs- und Verhaltensempfehlungen meines Arztes oder Therapeuten überprüfen.<br><br>…Behandlungsmöglichkeiten zusätzlich zu ärztlichen Empfehlungen finden.<br><br>…mir selbst bei einem akuten Gesundheitsproblem helfen.<br><br>Informational support<br><br>…über gesundheitliche Risiken und Krankheiten allgemein besser informiert sein mir selbst bei einem akuten Gesundheitsproblem helfen<br><br>…konkrete Informationen zur medizinischen Versorgung, zu Krankenkassen usw. finden<br><br>…die qualitativ besten Behandlungsmöglichkeiten oder medizinischen Einrichtungen finden.<br><br>…Tipps und Hilfen für eine gesündere Lebensweise finden.<br><br>…meine Familie oder Freunde bei gesundheitlichen Problemen beraten.<br><br>Emotional support<br><br>mit meinen Problemen nicht alleine sein, persönliche Unterstützung von anderen bekommen<br><br>Erfahrungen und Meinungen zu Gesundheitsfragen mit anderen austauschen
Education	Which level of school education do you have?<br><br>(different levels of German school education, representing 8,10,12 years	Welchen Schulabschluss haben Sie?<br><br>(Haupt-/ Volksschulabschluss, weiterführende Schule ohne Abitur,

	of school and university)	Mittlere Reife, Fach-/ Hochschulreife ohne Studium Fach-/ Hochschulreife mit Studium)
Occupation	Are you employed at the moment? What applies to you?<br><br>(fully employed, partly employed, education, not employed)	Sind Sie zurzeit erwerbstätig? Was auf dieser Liste trifft auf Sie zu? (vollzeit erwerbstätig, teilzeit erwerbstätig, in Ausbildung, nicht erwerbstätig)
Income	Net income of the household	HH-Nettoeinkommen
Household Size	Number of people in the household	Anzahl der Haushaltsmitglieder
